# Population pharmacokinetics modeling of albendazole and its active metabolite, albendazole sulfoxide in mass drug administration against lymphatic filariasis

**DOI:** 10.1128/aac.00562-26

**Published:** 2026-10-08

**Authors:** Abdullah Alshehri, Christopher L. King, Daryl J. Murry

**Affiliations:** 1 Department of Clinical Pharmacy, College of Pharmacy, King Khalid University 48144, Abha, Saudi Arabia; 2 Center for Global Health and Diseases, Department of Pathology, Case Western Reserve University 242911, Cleveland, Ohio, USA; 3 Veterans Affairs Research Service, VA Medical Center 20083, Cleveland, Ohio, USA; 4 Department of Pharmacy Practice and Science, University of Nebraska Medical Center 12284, Omaha, Nebraska, USA; 5 Fred and Pamela Buffett Cancer Center, University of Nebraska Medical Center 602161, Omaha, Nebraska, USA; University of Houston, Houston, Texas, USA

**Keywords:** population pharmacokinetics, bancroftian filariasis, lymphatic filariasis, mass drug administration, albendazole, albendazole sulfoxide

## Abstract

Albendazole (ALB), a benzimidazole antiparasitic, is widely used in mass drug administration (MDA) campaigns against lymphatic filariasis (LF). It is rapidly metabolized to its active form, albendazole sulfoxide (ALB-OX), which exhibits considerable interindividual variability (IIV) in pharmacokinetics (PK). This variation may affect ALB efficacy in MDA programs. We developed a population pharmacokinetic (PopPK) model characterizing ALB and ALB-OX exposure and identifying demographic and clinical covariates contributing to IIV in patients with LF and healthy individuals. Plasma concentration-time data were pooled from three clinical studies involving ALB co-administered with ivermectin and diethylcarbamazine. Nonlinear mixed-effects modeling was conducted using Phoenix NLME. Covariate effects (e.g., sex, age, infection status) were assessed, and model validation employed visual predictive checks and bootstrapping. Simulations explored covariate impact on exposure. A joint two-compartment model with transit absorption for ALB and pre-systemic ALB-OX formation best described the data. Sex significantly influenced ALB-OX clearance and volume of distribution, with males exhibiting higher clearance and lower exposure. Body weight, age, and LF infection status did not significantly affect PK parameters. Simulations confirmed 33% lower ALB-OX levels in males compared to females following a 400 mg oral ALB dose. This is the first PopPK model of ALB and ALB-OX in LF-endemic populations. It highlights sex as a key covariate explaining IIV in ALB-OX exposure, with implications for optimized dosing in MDA programs. Further studies incorporating pharmacogenetics and pediatric data are warranted.

This study is registered with ClinicalTrials.gov as NCT04410406, NCT02845713, and NCT03664063.

## INTRODUCTION

Albendazole (ALB) is a benzimidazole derivative antiparasitic drug with broad-spectrum activity against various parasitic and helminth infections (1). Discovered in 1970, it has a wide range of therapeutic uses in both human and veterinary medicine (2). ALB is also employed as preventive chemotherapy, either alone or in combination with other drugs, in mass drug administration (MDA) campaigns targeting neglected tropical diseases (3). It is commonly administered as a single oral dose of 400 mg as part of MDA programs for the prevention and elimination of lymphatic filariasis (LF). Following oral administration, ALB undergoes extensive first-pass hepatic metabolism and is rapidly converted to albendazole sulfoxide (ALB-OX), the pharmacologically active metabolite responsible for its antiparasitic activity. ALB-OX is subsequently metabolized to the inactive metabolite albendazole sulfone. Due to extensive first-pass metabolism, systemic exposure to the parent drug is limited, with an elimination half-life of approximately 1–2 h, whereas ALB-OX has a longer elimination half-life of approximately 7–8 h and accounts for the majority of systemic antiparasitic activity (4).

Several studies have characterized the pharmacokinetics (PK) of ALB in humans after a single oral dose, and high interindividual variability (IIV) in drug exposure and PK were reported for the parent drug and ALB-OX (5–9). Furthermore, several studies have evaluated the PK of ALB and ALB-OX as part of the ivermectin, diethylcarbamazine, and albendazole (IDA) treatment protocol against LF and found substantial IIV in the systemic exposure of ALB-OX (10–12). For example, after a single 400 mg oral dose of ALB as part of the IDA protocol, the drug exposure parameters were estimated with significantly high IIV for ALB-OX, with coefficients of variation (CV%) of the estimated area under the plasma concentration–time curve (AUC) and maximum plasma concentration (C_max_) of 80.5% and 81.9%, respectively (13). Moreover, ALB-OX exposure was shown to vary by age and infection type (14). Identifying the sources of high IIV in the PK of ALB and ALB-OX, and quantifying their impact on overall exposure, is essential for effective and safe dosing. Population pharmacokinetic (PopPK) analysis can provide the necessary tools to evaluate PK in a specific population and identify the sources of IIV (15, 16).

This study aims to characterize the PopPK of ALB and ALB-OX in patients with LF and healthy individuals using nonlinear mixed-effects (NLME) modeling and investigate the impact of demographic and clinical characteristics on IIV.

## MATERIALS AND METHODS

### Clinical studies

Plasma concentration-time profiles and demographic and clinical characteristics were available for modeling from three published PK studies (NCT02845713, NCT04410406, NCT03664063) aimed to assess the PK of the IDA chemotherapy protocol in LF patients and healthy individuals (10, 11, 13). In all three studies, subjects received a single oral dose of ALB (400 mg) with ivermectin and diethylcarbamazine after a fatty breakfast. In the NCT04410406 and NCT03664063 studies, ALB was co-administered with a single oral dose of moxidectin or azithromycin, respectively, in select subjects as outlined in the protocol. A summary of the studies, including plasma sampling times after oral dosing, is shown in Table 1. The plasma samples were analyzed using a previously reported liquid chromatography–mass spectrometry (LC-MS) method with lower limits of quantification (LLOQ) for ALB and ALB-OX of 0.1 ng/mL and 0.5 ng/mL, respectively (17, 18).

**TABLE 1:** Summary of clinical trials^*a*^

Study	Design/region	ALB dose	Number of subjects who received ALB	Plasma sampling (h)
Pharmacokinetic and safety study of co-administration of IDA and azithromycin for the integrated treatment of NTDs	open-label, randomized, 3-arm, Papua New Guinea	400 mg	Healthy adults (*n* = 26)	0, 1, 2, 3, 4, 6, 8, 12, 24, 48, 72
Pharmacokinetics, safety, and efficacy of a single co-administered dose of IDA in adults with and without *Wuchereria bancrofti* infection in Côte d’Ivoire	open-label cohort study, single-arm, Côte d’Ivoire	400 mg	IDA treatment naïve LF-infected (*n* = 32) and uninfected (*n* = 24) adults	0, 1, 2, 3, 4, 6, 8, 12, 24, 36, 48, 72, 168
Safety and efficacy of moxidectin combination treatments vs ivermectin combination treatments for bancroftian filariasis	open-label, randomized, 4-arm, Côte d’Ivoire	400 mg	LF patients (*n* = 58)	0, 2, 3, 4, 6, 12, 24, 48, 72, 168

^*a*^ ALB, albendazole; NTDs, neglected tropical diseases; LF, lymphatic filariasis.

### Population pharmacokinetic modeling

The PopPK modeling was carried out using Phoenix NLME software (version 8.4; Certara L.P., St. Louis, MO, USA). The first-order conditional estimation with extended least squares algorithm (FOCE-ELS) was employed as the model engine to estimate the fixed-effect parameters of the model, along with IIV and remaining unexplained errors. The model-building process began with examining different base models to describe the drug exposure of the parent drug (ALB). Various structural models with one, two, and three drug disposition compartments were assessed in combination with different error models. Additionally, drug absorption post-dosing was investigated using different scenarios, including absorption delay scenarios such as the Tlag function and transit compartment models.

After selecting the best base model describing ALB drug exposure, the ALB-OX model was built by connecting it to the established ALB base model. ALB systemic clearance was used as the metabolite formation parameter, with a formation ratio fixed to one. Several structural and error models were used to evaluate ALB-OX exposure and associated IIV. Several scenarios for the joint model were assessed, including sequential and simultaneous fitting. In sequential modeling, the fixed and random parameters of the ALB base model were fixed before adding the ALB-OX model. In contrast, in simultaneous modeling, they were estimated jointly for both compounds. Allometric scaling to describe the impact of body weight on volume of distribution (VD) and clearance (CL) for ALB and ALB-OX was also evaluated (19). Plasma samples below LLOQ for both compounds were censored using the M3 or M5 methods, and model fitting was assessed by including the actual reported concentrations (20, 21). The covariate analysis was performed after selecting the base joint model to evaluate the possible impact of demographic and clinical characteristics on the reported high IIV of ALB and ALB-OX PK. The tested covariates included age, sex, weight, alanine aminotransferase (ALT), aspartate aminotransferase (AST), plasma creatinine, co-administration of moxidectin or azithromycin, and LF infection status. Continuous covariates were incorporated into the model using a power function normalized to the median value of each covariate in the study population:


(1) $$P_{i}=P_{TV}{\left(\frac{Cov_{i}}{Cov_{median}}\right)}^{\theta_{cov}}\exp\left(\eta_{i}\right)$$

*where* $P_{i}$ is the individual parameter estimate, $P_{TV}$ is the typical population parameter value, $Cov_{i}$ is the individual covariate value, $Cov_{median}$ is the median value of the covariate in the study population, $\theta_{cov}$ is the estimated covariate effect, and $\eta_{i}$ is the interindividual random effect.

Categorical covariates were incorporated into the model using indicator (dummy-coded) variables according to the following equation:


(2) $$P_{i}=P_{TV}\exp\left(C\theta_{cov}\times Cov_{cat}\right)\exp\left(\eta_{i}\right)$$

*where* $Cov_{cat}$ is the categorical covariate coded as 0 for the reference category and one for the comparison category, and all other terms are as previously defined.

Covariate effects were evaluated using a stepwise covariate modeling approach consisting of forward inclusion followed by backward elimination. During the forward inclusion step, each covariate was added individually to the base model, and the resulting objective function value (OFV) was compared with that of the base model. A covariate was considered statistically significant and retained in the full model if its inclusion resulted in a reduction in OFV greater than 3.84, corresponding to a significance level of *P* < 0.05 for one degree of freedom. Covariates retained in the full model were subsequently evaluated using backward elimination and were retained in the final model if their removal resulted in an increase in OFV greater than 6.63, corresponding to a significance level of *P* < 0.01 for one degree of freedom. Covariates meeting these criteria were considered to significantly explain IIV associated with the estimated PK parameters. The selection of the final model was guided by goodness-of-fit (GOF) criteria, including the reduction in OFV, visual inspection of diagnostic plots describing the model’s prediction and error distributions, and the model’s ability to estimate parameters with acceptable precision. The final selected model was validated using internal validation methods, including visual predictive check (VPC) and bootstrapping.

### Simulation

The final model was used to simulate plasma concentration-time profiles of ALB-OX after a single 400 mg oral dose of ALB in 200 virtual subjects to further evaluate the influence of significant covariates on drug exposure parameters. The virtual subjects were generated by sampling from the original study population used for model development. Consequently, the demographic characteristics and covariate distributions (e.g., age, sex, body weight, and other covariates) of the simulated subjects reflected those observed in the study population. The simulation was conducted using Monte Carlo simulation, and noncompartmental analysis (NCA) was performed using PKanalix software (2023R1, Lixoft SAS, a Simulations Plus company) to estimate the area under the plasma concentration–time curve from time zero to the last measurable concentration (AUC_last_) and C_max_ parameters of simulated plasma concentration profiles.

## RESULTS

### Population pharmacokinetic analysis

**Fig 1:**
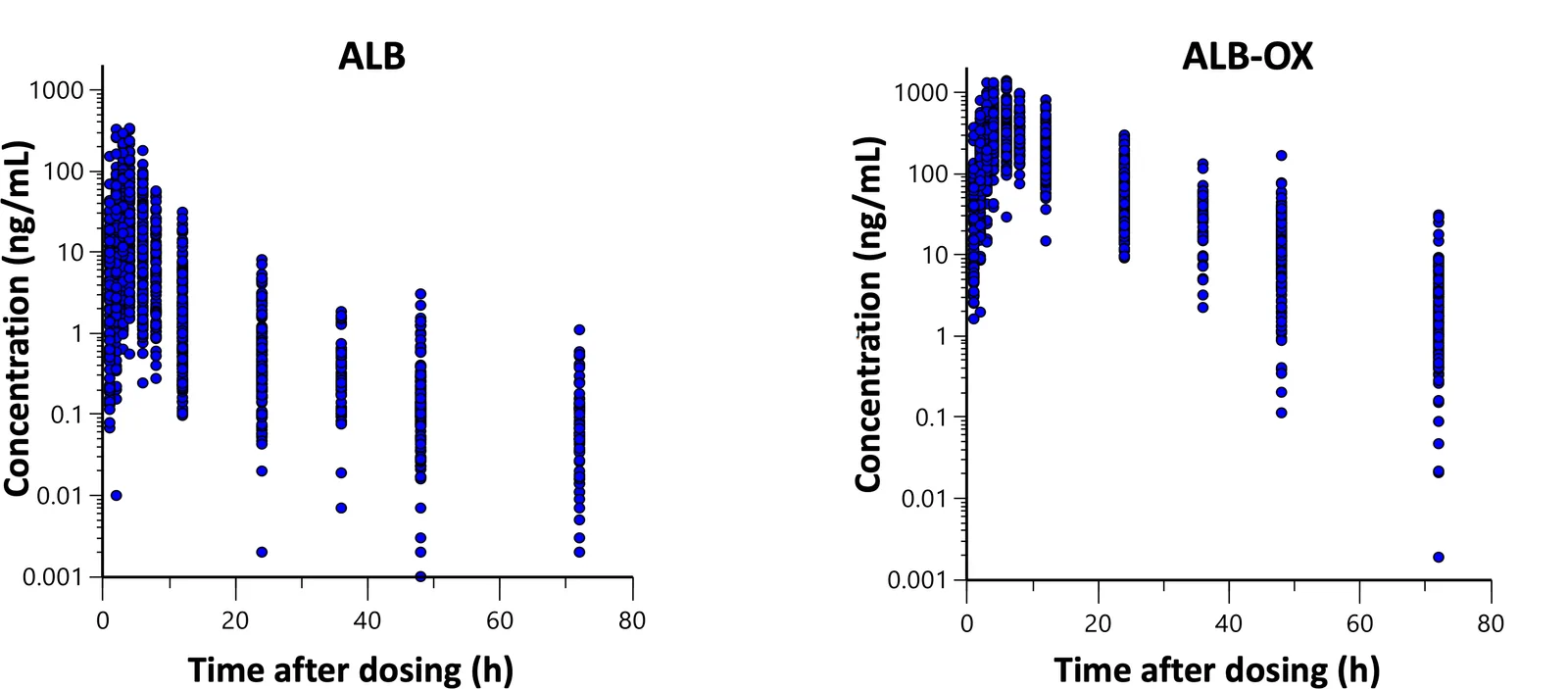
Albendazole and albendazole sulfoxide plasma concentration vs time following a single oral dose of ALB (400 mg).

**Fig 2:**
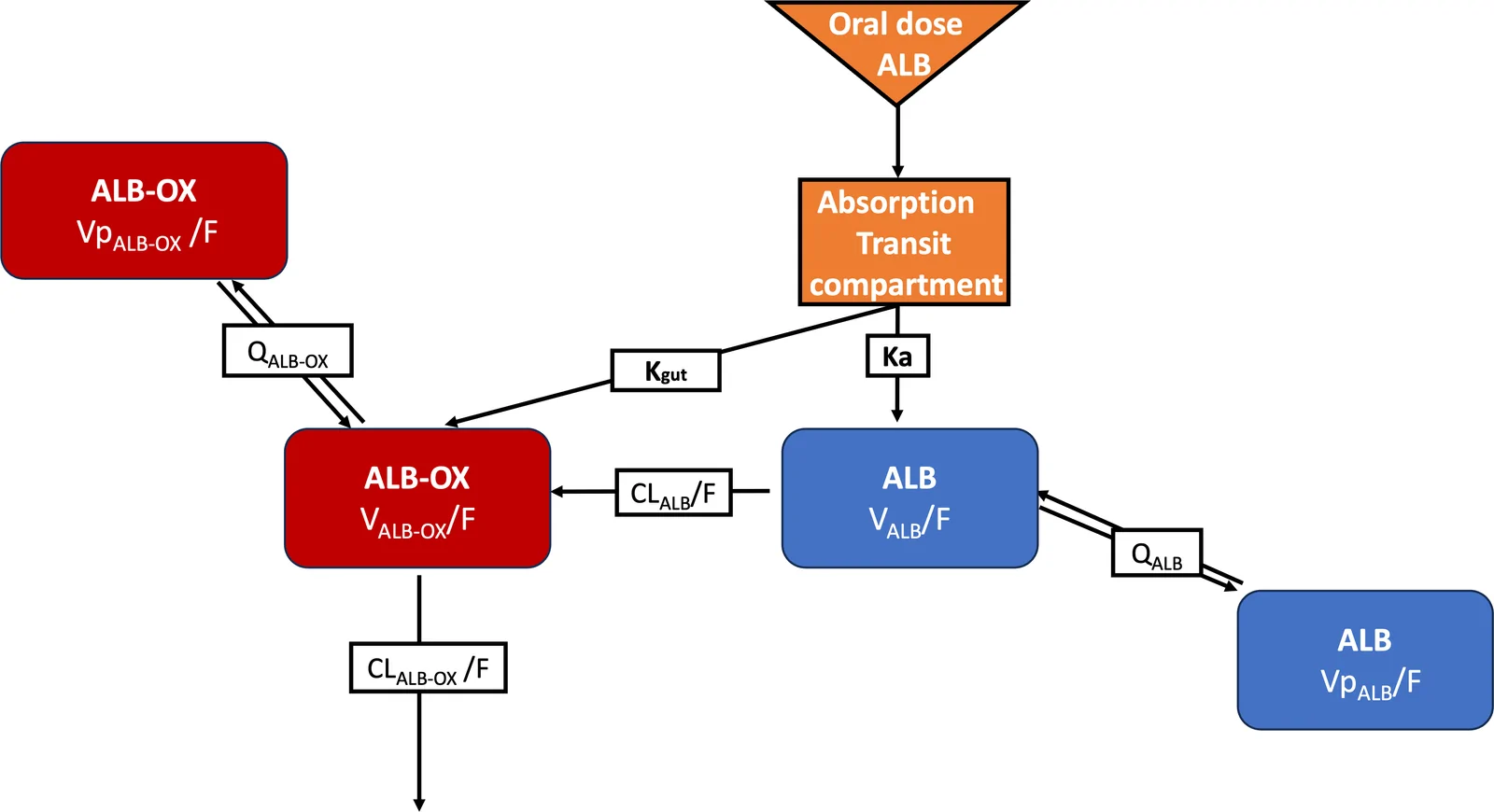
Schematic description of the structural model. V_ALB_/F, central apparent volume of distribution of ALB; Vp_ALB_/F, peripheral apparent volume of distribution of ALB; CL_ALB_/F, apparent clearance of ALB; Q_ALB_, intercompartmental clearance of ALB; K_a_, absorption rate constant; K_gut_, ALB-OX gut pre-metabolism formation rate; V_ALB-OX_/F, central apparent volume of distribution of ALB-OX; Vp_ALB-OX_/F, peripheral apparent volume of distribution of ALB-OX; CL_ALB-OX_/F, apparent clearance of ALB-OX; Q_ALB-OX_, intercompartmental clearance of ALB-OX

A total of 2,545 plasma samples from 140 subjects were available for model building. The plasma concentration-time profiles of ALB and ALB-OX in study subjects are presented in Fig. 1. The demographic and clinical characteristics of the study subjects are summarized in Table 2. A two-compartment model with transit absorption into the central compartment and linear elimination best described ALB exposure after a single oral dose. The transit absorption compartment model showed superiority in explaining the delay in ALB absorption after oral dosing compared to other absorption scenarios, including Tlag and parallel zero- and first-order absorption. As a result, it was incorporated into the model. For ALB-OX, drug exposure was best described by a two-compartment model with linear metabolite formation and elimination. A proportional-error model best described the remaining unexplained variability for ALB, whereas a combined-error model was used for ALB-OX. A schematic description of the joint structural model of ALB and ALB-OX is provided in Fig. 2.

**TABLE 2:** Summary of subject demographics and clinical characteristics^*a*^

Demographic and clinical characteristics	Value
Sex	
No. of males (%)	98 (70%)
No. of females (%)	42 (30%)
Infection status	
Lymphatic filariasis −ve (%)	50 (36%)
Lymphatic filariasis +ve (%)	90 (64%)
Geographic area	
Côte d'Ivoire (%)	114 (81%)
Papua New Guinea (%)	26 (19%)
Age (yr) Median (range)	35 (18–66)
Weight (kg) Median (range)	60.45 (46–135)
ALT (U/L) Median (range)	26 (12–67)
AST (U/L) Median (range)	30 (15–73)
Serum creatinine (mg/dL) Median (range)	1 (0.3–1.7)

^*a*^ ALT, alanine transaminase; AST, aspartate aminotransferase.

The ALB and ALB-OX models were initially linked only by the systemic metabolite formation parameter, represented by the ALB total clearance (CL_ALB_/F). However, the resulting joint model showed structural misspecification, as confirmed by visual inspection of the diagnostic plot depicting the relationship between conditional weighted residuals (CWRES) and time after dosing. To describe the amount of ALB-OX formed in the gut epithelium before the first-pass metabolism of ALB (pre-systemic formation), a model parameter was added to account for this part of ALB metabolism (K_gut_). Adding the K_gut_ parameter to the joint model was associated with a significant reduction in OFV (ΔOFV = −687) compared to the model without pre-systemic ALB-OX formation; it also corrected the structural bias observed previously in the CWRES vs time plot.

Simultaneous fitting for ALB and ALB-OX was used in the joint model, as sequential fitting resulted in a significant increase in OFV (ΔOFV = 515). To account for the difference in molecular mass between ALB and ALB-OX, the plasma concentration of ALB-OX in the study subjects was adjusted by 0.943, which is the molecular mass ratio of the two compounds. For plasma samples below LLOQ, the actual reported values were used in the modeling, as their inclusion improved model predictions compared with recommended censoring methods. Application of the M3 and M5 censoring methods did not improve model performance and was associated with persistent underprediction of the lower 5th percentile of ALB plasma concentrations. Furthermore, the M3 model exhibited estimation instability, preventing reliable estimation of standard errors, confidence intervals, and parameter precision (CV%).

**Fig 3:**
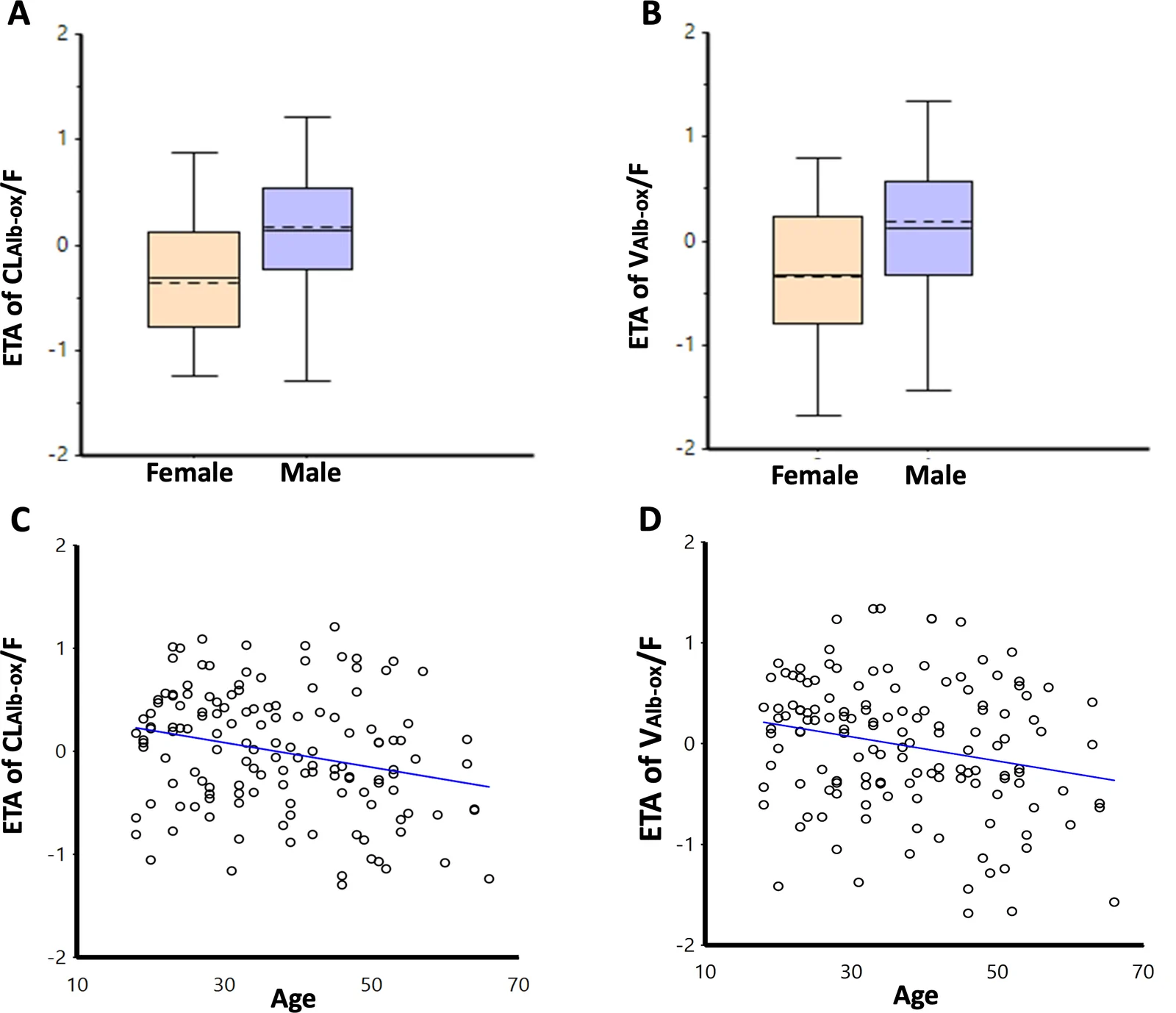
ETA Box Plots. Box plots (**A and B**) and plots (**C and D**) illustrate the impact of sex and age on ETA values of CL_ALB-OX_/F and V_ALB-OX_/F. ETA, variance of interindividual variability; CL_ALB-OX_/F**,** apparent clearance of ALB-OX; V_ALB-OX_/F, central apparent volume of distribution of ALB-OX

**Fig 4:**
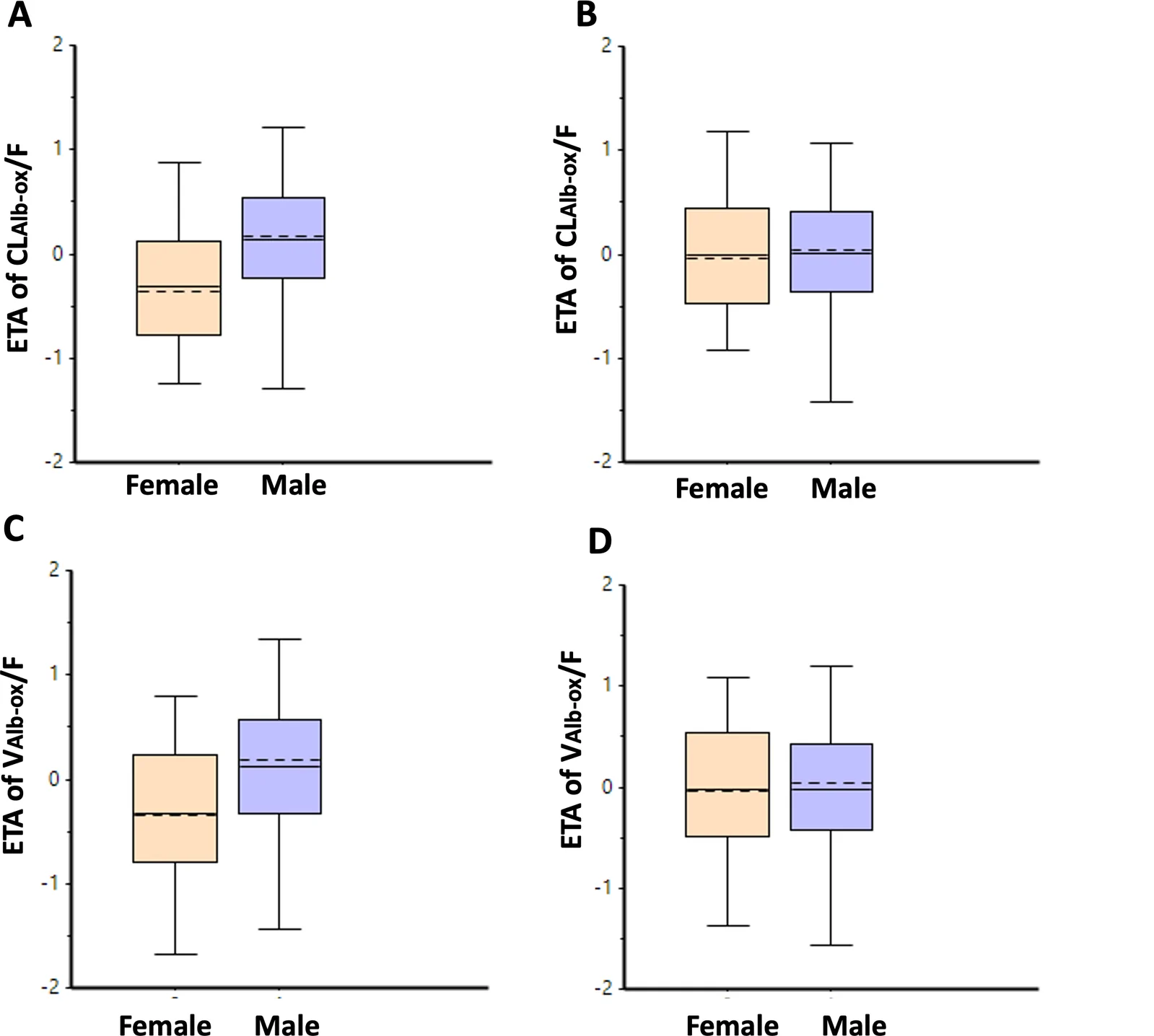
ETA plots for sex effect on apparent clearance and volume of distribution of albendazole sulfoxide. Box plots show the effect of sex on ETA values of CL_ALB-OX_/F and V_ALB-OX_/F in the base model (**A and C**) and after incorporating its effect in the final model (**B and D**). ETA, variance of interindividual variability; CL_ALB-OX_/F, apparent clearance of ALB-OX; V_ALB-OX_/F, central apparent volume of distribution of ALB-OX

**Fig 5:**
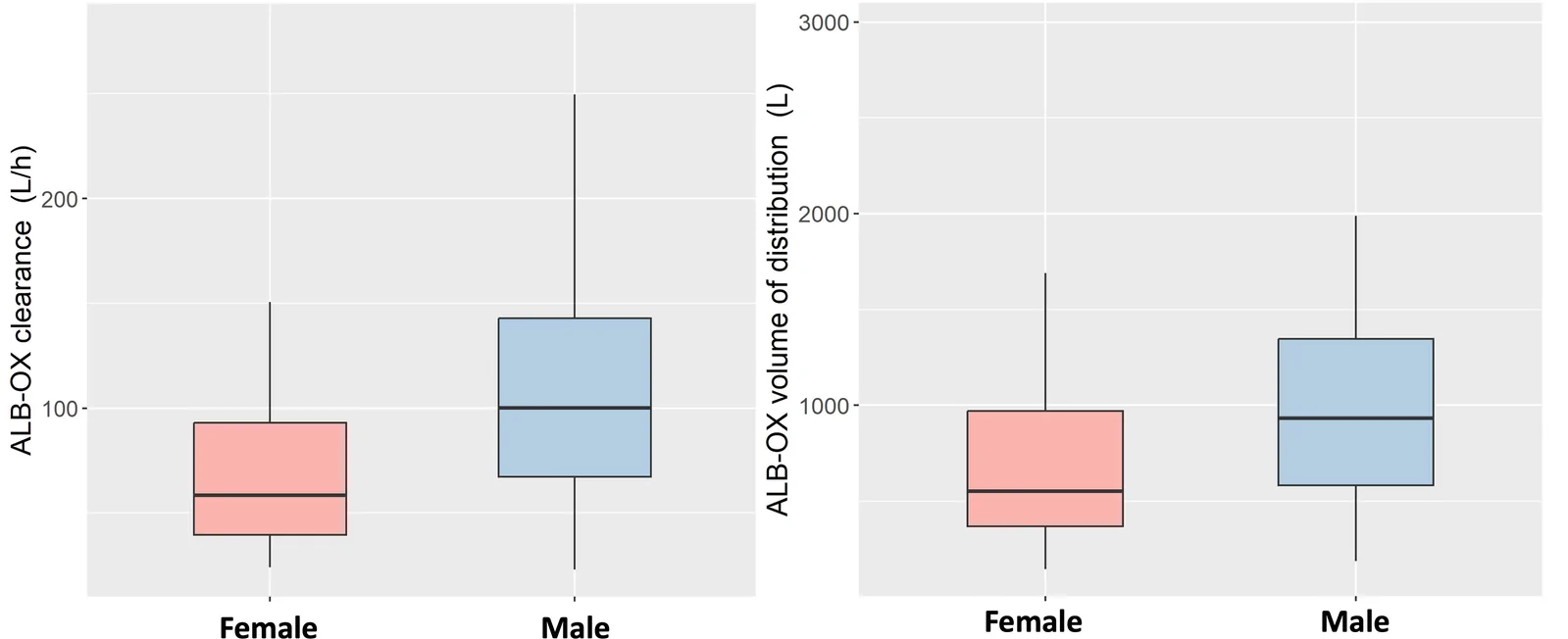
Sex impact on the final model post hoc estimates of apparent clearance and apparent volume of distribution of albendazole sulfoxide.

Visual inspection of empirical Bayes estimates (ETA) plots showed some correlation between ETA values and several covariates, such as sex and age, which were further investigated for significance using forward addition and backward elimination (Fig. 3). Covariate analysis showed that sex was a significant covariate explaining part of the IIV in the apparent clearance (CL_ALB-OX_/F) and central apparent volume of distribution (V_ALB-OX_/F) of ALB-OX (*P* < 0.05). Compared to the base model, incorporating the sex effect in the final model decreased random IIV in CL_ALB-OX_/F and V_ALB-OX_/F by 12% and 9%, respectively. Moreover, it corrected the observed correlations in the base model’s ETA plots of CL_ALB-OX_/F and V_ALB-OX_/F (Fig. 4). The post hoc analysis revealed that male subjects had higher estimated CL_ALB-OX_/F and V_ALB-OX_/F values than female subjects (Fig. 5). The final model-estimated mean values for CL_ALB-OX_/F in women and men were 61.2 L/h and 95 L/h, respectively. For V_ALB-OX_/F, the estimated values were 569.5 L for women and 884 L for men. The following equations show the relationship between sex and CL_ALB-OX_/F and V_ALB-OX_/F in the final model:


$$\begin{array}{rl} & {CL}_{ALB-OX}/F=61.2\cdot e^{0.44\times \mathrm{Sex}(0=\text{ female },1=\text{ male })}\\ & V_{ALB-OX}/F=569.5\cdot e^{0.44\times \mathrm{Sex}(0=\text{ female },1=\text{ male })} \end{array}$$

Although ETA plots also revealed that age affected ETA values, with increasing age associated with a downward trend in the variance of CL and VD for ALB-OX, this effect was not statistically significant in the covariate analysis. In our study population, body weight did not explain IIV associated with the CL and VD parameters of ALB and ALB-OX by either allometric scaling or covariate analysis.

**Fig 6:**
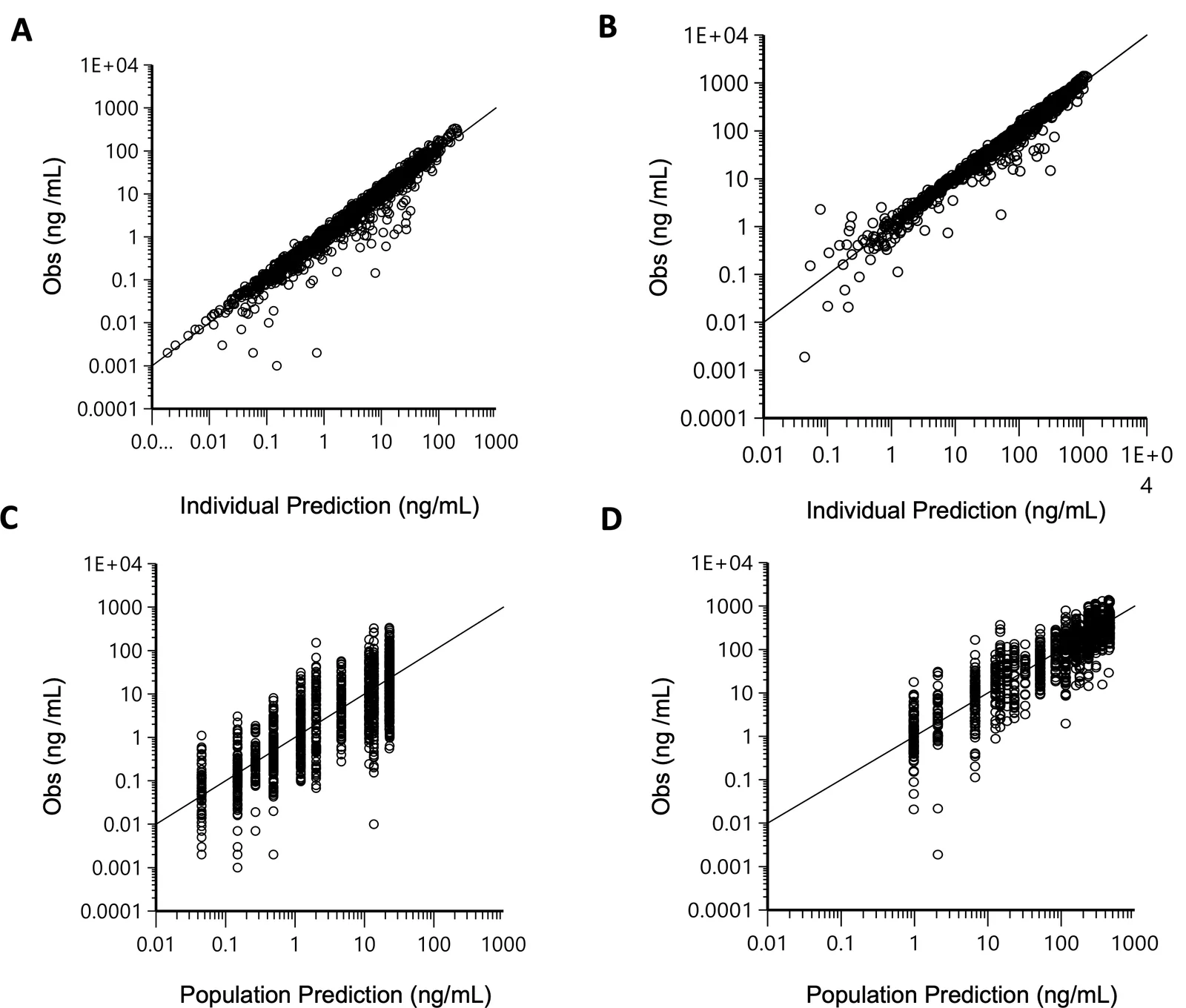
Goodness-of-fit (GOF) plots for the final joint population pharmacokinetic model. (**A**) Observed versus individual-predicted (IPRED) concentrations for albendazole (ALB). (**B**) Observed versus individual-predicted (IPRED) concentrations for albendazole sulfoxide (ALB-OX). (**C**) Observed versus population-predicted (PRED) concentrations for ALB. (**D**) Observed versus population-predicted (PRED) concentrations for ALB-OX. The solid diagonal line represents the line of identity.

**Fig 7:**
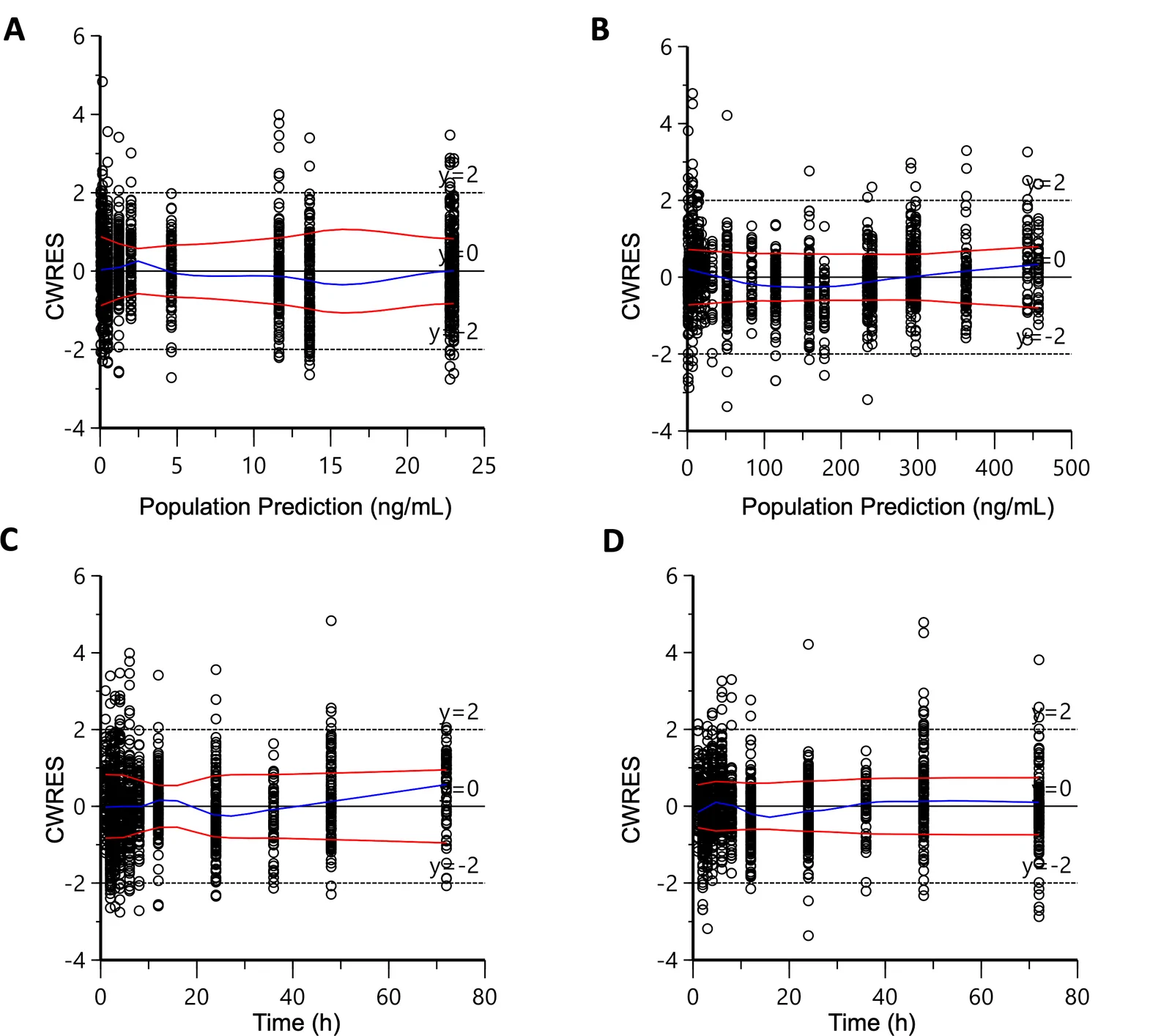
Conditional weighted residual (CWRES) diagnostic plots for the final joint population pharmacokinetic model. (**A**) CWRES versus population-predicted (PRED) concentrations for albendazole (ALB). (**B**) CWRES versus population-predicted (PRED) concentrations for albendazole sulfoxide (ALB-OX). (**C**) CWRES versus time for ALB. (**D**) CWRES versus time for ALB-OX. The solid horizontal line indicates a residual value of zero, and the dashed horizontal lines represent CWRES values of ±2. The blue line represents the locally weighted scatterplot smoothing (LOESS) trend, while the red lines indicate the corresponding 95% confidence intervals.

The final model estimates of PK parameters and associated random IIV are summarized in Table 3. The final model showed acceptable precision in estimating the PK parameters of the joint model. The random IIV for all PK parameters was estimated in the final model, except for V_ALB_/F and Q_ALB-OX_, for which the estimates were associated with high shrinkage. Visual inspection of diagnostic plots revealed good agreement between model individual and population predictions (IPRED, PPRED) and observations (Fig. 6). The CWRES distribution exhibited a symmetrical and even distribution around the zero line post-dosing and across the population concentration range of ALB and ALB-OX, demonstrating the model’s ability to explain the residual unexplained variability (Fig. 7).

**TABLE 3:** Final model estimates of population PK parameters of albendazole and albendazole sulfoxide^*a*^

Parameter	Mean value estimate (CV%)	Shrinkage (%)	Bootstrap median value (2.5th-97.5th percentiles)
Fixed–effect parameters			
V_ALB_/F (L)	550.4 (22)		549.4 (360–894.6)
Vp_ALB_/F (L)	976.2 (23)		1,001.2 (629.5–1,719.2)
CL_ALB_/F (L/h)	267 (21)		268.2 (187.4–431.3)
Q_ALB_ (L/h)	60.6 (24)		61.6 (44.5–103.2)
Ka (1/h)	0.14 (46)		0.15 (0.09–0.28)
MTT (h)	2.2 (9)		2.2 (2–2.4)
NTC	3 (12)		3 (2.3–3.6)
K_gut_ (1/h)	1.5 (38)		1.6 (1–2.2)
V_ALB-OX_/F (L)	569.5 (16)		554.3 (448.9–666.7)
Vp_ALB-OX_/F (L)	108.5 (22)		109.6 (71.2–160.8)
CL_ALB-OX_/F (L/h)	61.2 (14)		60.4 (51.2–70.3)
Q_ALB-OX_ (L/h)	20.3 (32)		20.4 (12.6–39.3)
Covariates–effect			
Sex (male) on CL_ALB-OX/F_	0.44 (29)		0.45 (0.27–0.63)
Sex (male) on V_ALB-OX/F_	0.44 (32)		0.46 (0.24–0.69)
Random–effect parameters			
V_ALB_/F	-	-	-
Vp_ALB_/F	0.54 (84)	18	0.52
CL_ALB_/F	0.05 (22)	35	0.05
Q_ALB_	0.56 (86)	16	0.53
Ka	1.2 (150)	19	1.17
MTT	0.13 (37)	13	0.12
NTC	0.96 (120)	18	0.94
K_gut_	0.68 (98)	34	0.72
V_ALB-OX_/F	0.4 (70)	3	0.40
Vp_ALB-OX_/F	0.4 (70)	34	0.38
CL_ALB-OX_/F	0.3 (59)	2	0.30
Q_ALB-OX_	-	-	-
Residual variability			
Proportional residual error (ALB)	0.57 (7)		0.55 (0.10–0.88)
Additive residual error (ALB-OX) (ng/mL)	0.43 (4)		0.43 (0.38–0.46)
Proportional residual error (ALB-OX)	0.23 (2)		0.23 (0.20–0.26)
Correlation (V_ALB-OX_/F, CL_ALB-OX_/F)	0.88		0.88

^*a*^ V_ALB_/F, central apparent volume of distribution of ALB; Vp_ALB_/F, peripheral apparent volume of distribution of ALB; CL_ALB_/F, apparent clearance of ALB; Q_ALB_, intercompartmental clearance of ALB; K_a_, absorption rate constant; MTT, mean transit time; NTC, number of transit compartments; K_gut_, ALB-OX gut pre-metabolism formation rate; V_ALB-OX_/F, central apparent volume of distribution of ALB-OX; Vp_ALB-OX_/F, peripheral apparent volume of distribution of ALB-OX; CL_ALB-OX_/F, apparent clearance of ALB-OX; Q_ALB-OX_, intercompartmental clearance of ALB-OX.

### Model evaluation

**Fig 8:**
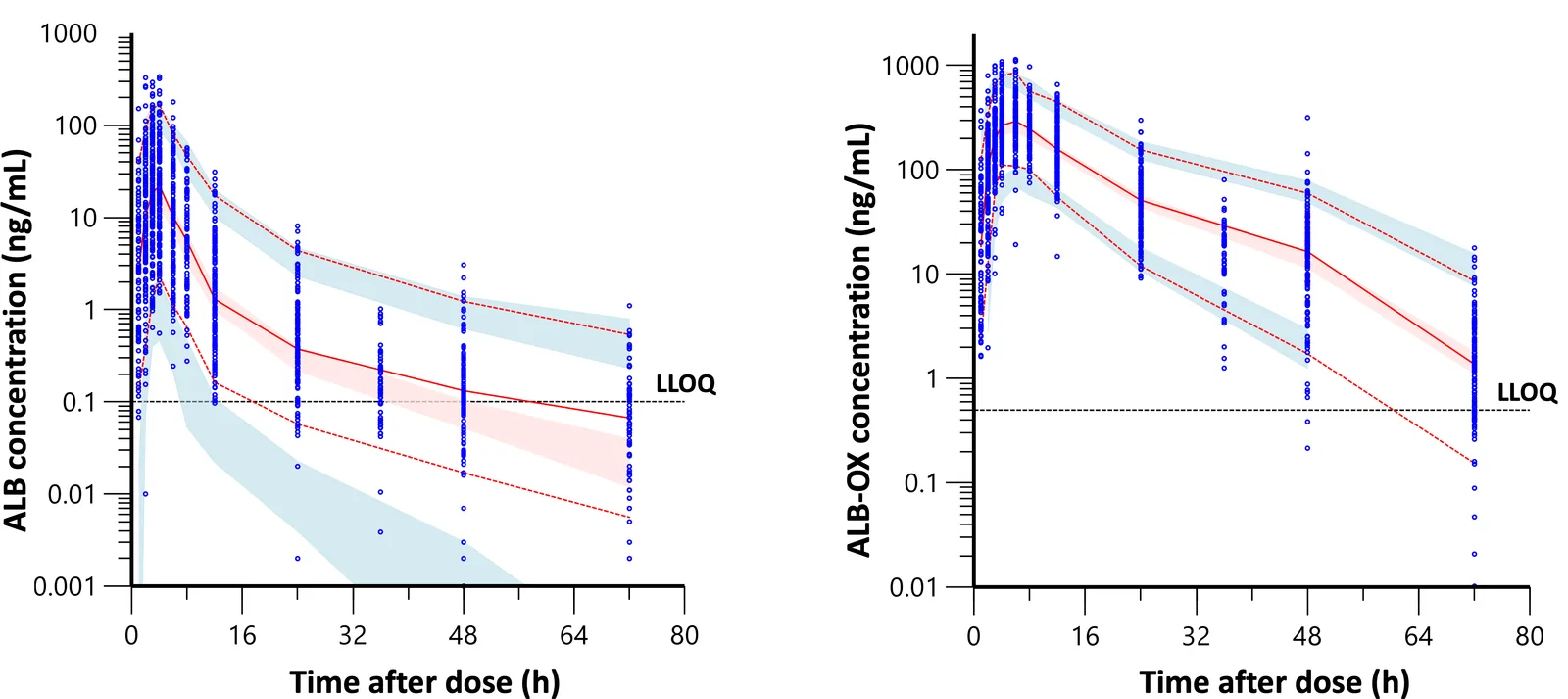
Visual predictive check of albendazole (left) and albendazole sulfoxide (right) following a single oral dose of ALB (400 mg). Solid red lines represent the observed data’s (blue dots) 50th percentile. Dashed red lines represent the 5th and 95th percentiles of observed data. Shaded areas (red and blue) show a 95% prediction interval of the 5th, 50th, and 95th simulated data (*n* = 1,000). Dashed black lines are lower limits of quantification for ALB (0.1) and ALB-OX (0.5).

**Fig 9:**
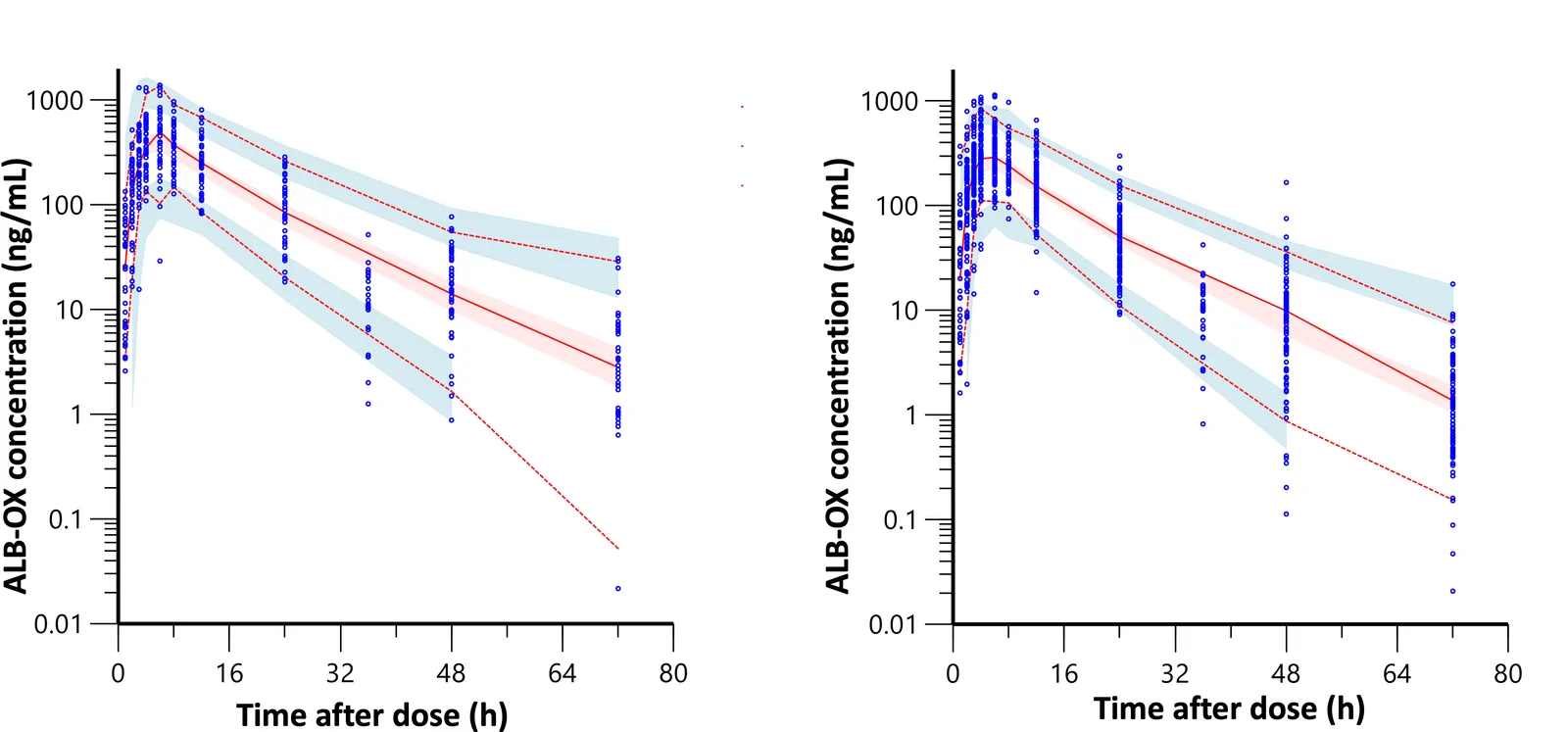
Visual predictive check stratified by sex of albendazole sulfoxide following a single oral dose of albendazole (400 mg) (left = female, right = male). Solid red lines represent the observed data’s (blue dots) 50th percentile. Dashed red lines represent the 5th and 95th percentiles of observed data. Shaded areas (blue and red) show a 95% prediction interval of the 5th, 50th, and 95th simulated data (*n* = 1,000).

Model evaluation by VPC demonstrated good prediction performance of the final model. For ALB-OX, the model demonstrated an excellent ability to characterize the central tendency of observed ALB-OX concentrations, with the median percentile of observed concentrations maintained well within the prediction interval of the simulated median percentile (Fig. 8). Moreover, the 10th and 90th percentiles of observed ALB-OX concentrations were maintained within the 95% prediction intervals of the corresponding percentiles from simulated data. The model showed overall acceptable prediction performance in predicting observed ALB concentrations; however, an underprediction of the lower 5th percentile of data were observed. The VPC for ALB-OX, stratified by sex, is presented in Fig. 9. The bootstrapping findings demonstrated good agreement between bootstrapping estimates of PK parameters and the final model estimations, indicating the robust performance of the final model (Table 3).

### Simulation

**Fig 10:**
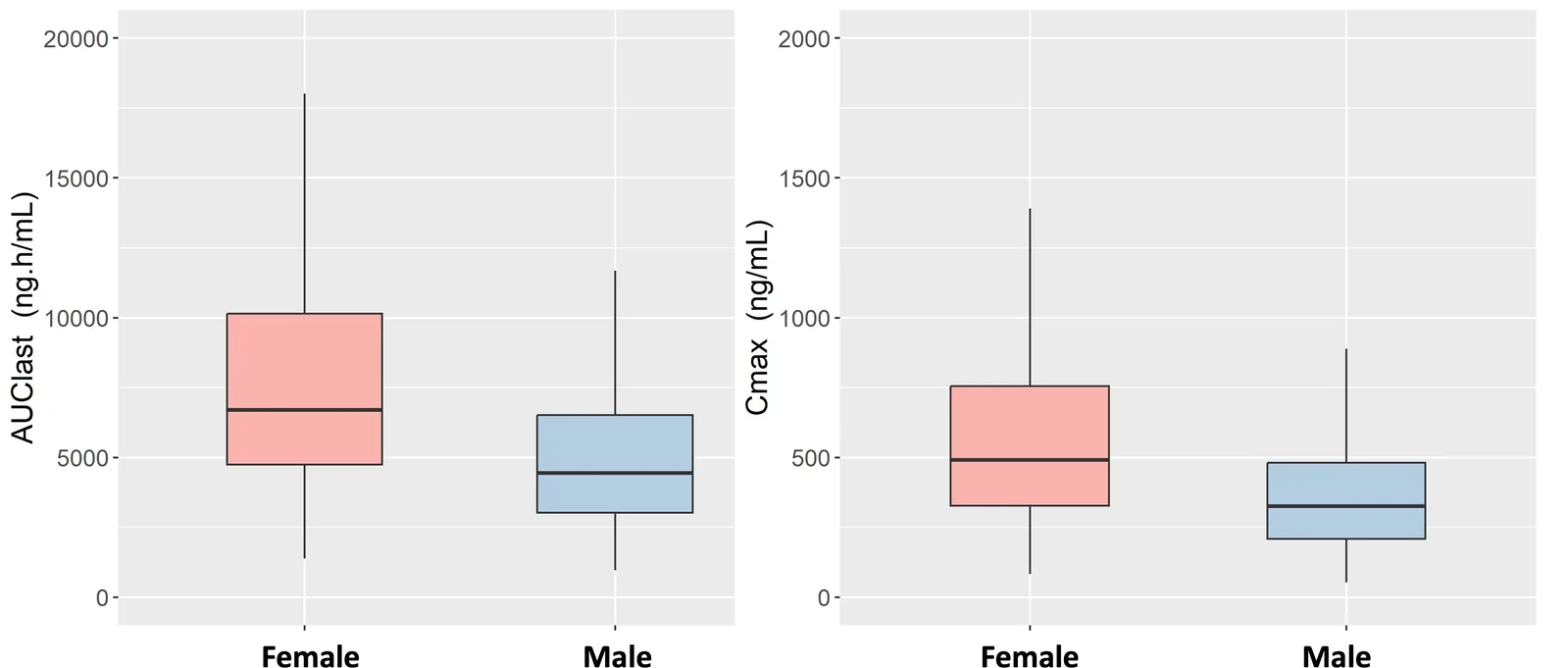
Comparison of model-based simulated albendazole sulfoxide exposure in virtual males and females after a single oral dose of 400 mg of albendazole. AUC_last_, area under the plasma concentration-time curve from 0 to the last quantifiable concentration; C_max_, maximum plasma concentration

Following a single 400 mg oral dose of ALB, the model-based simulation showed that ALB-OX exposure was lower in males, with AUC_last_ and C_max_ being 33.6% and 33.7% lower in males than females (Fig. 10). The model-based median simulated AUC_last_ and C_max_ of ALB-OX stratified by sex after a single 400 mg oral dose of ALB are summarized in Table 4.

**TABLE 4:** Model-based median (IQR) simulated AUC_last_ and C_max_ of albendazole sulfoxide stratified by sex after a single oral dose of ALB (400 mg)^*a*^

PK parameters	Median (IQR)	
	Male	Female
AUC_last_ (ng.h/mL)	4,446.94 (3,028.68–6,521.76)	6,699.3 (4,736.27–10,139.97)
C_max_ (ng/mL)	325.6 (209.09–481.85)	491.7 (327.38–756.22)

^*a*^ AUC_last_, area under the plasma concentration-time curve from 0 to the last quantifiable concentration; C_max_, maximum plasma concentration; IQR, interquartile range.

## DISCUSSION

In this analysis, we developed a PopPK model to describe ALB and ALB-OX PK and the related IIV in healthy individuals and patients with LF. Previous studies have reported modeling results from patients with neurocysticercosis and soil-transmitted helminth infections (22, 23). Our model is based on drug exposure data from LF patients and healthy subjects residing in LF-endemic areas. To the best of our knowledge, this is the first PopPK analysis to evaluate the PK of ALB and ALB-OX in LF MDA-eligible populations.

After oral dosing, ALB is rapidly metabolized to ALB-OX primarily by the first-pass effect in the liver. It has been shown that ALB-OX metabolic formation can occur in the gut before first-pass hepatic metabolism; however, previously published PopPK models of ALB and ALB-OX did not account for this pre-systemic ALB-OX formation (24, 25). In our analysis, we initially assessed the fit of various structural models in which ALB systemic metabolism was the only source of ALB-OX formation in the joint model. However, all tested models showed similar misspecification in the CWRES vs time plot, indicating underprediction of ALB-OX plasma concentrations in study subjects. Adding a model parameter to describe the pre-systemic metabolic formation of ALB-OX improved the fit of the joint model and the model’s ability to predict ALB-OX plasma concentrations. The final model estimates of ALB-OX PK parameters were consistent with previously reported estimates from NCA analysis; however, our final model showed lower estimates of VD and CL for ALB (10, 11). A possible explanation for this finding is that we accounted for the amount of ALB metabolized in the gut into ALB-OX, which might lead to a lower estimation of ALB total exposure in our model.

ALB is classified as a class II biopharmaceutical compound with very low solubility and high permeability (26). The limited solubility and other physiological factors impacting bioavailability might play a role in the high IIV observed with ALB and ALB-OX. In class II biopharmaceutical compounds, the dissolution rate is a determinant factor of drug bioavailability (27, 28). In our analysis, we assessed various models with different absorption scenarios to determine the impact of dissolution rate on ALB and ALB-OX PK, particularly absorption-related parameters. The tested models included inverse Gaussian and Weibull models with distribution-related parameter estimates derived from previously published physiologically based pharmacokinetic (PBPK) models (29). However, these models did not improve the joint model fit. The transit absorption compartment model best described the absorption of ALB in our final model and was associated with an overall improvement in model fit. Another impact of the dissolution rate on ALB PK is dose-dependent bioavailability, where increasing ALB dose was associated with reduced systemic exposure to ALB-OX (30). The drug exposure data in our analysis were derived from administering ALB as a single oral dose of 400 mg in all three clinical trials used to develop the model. As a result of the fixed oral dosing, we could not evaluate the effect of dose variations on ALB bioavailability and its consequent impact on the PK of ALB and ALB-OX.

One of the main objectives of this study was to assess the role of demographic and clinical related factors in explaining the reported high IIV associated with the PK of ALB and ALB-OX. The covariate analysis revealed that sex was statistically significant in explaining part of the IIV associated with CL_ALB-OX_/F and V_ALB-OX_/F. The post hoc analysis showed that the model estimates for systemic clearance of ALB-OX and distribution in females were lower than the estimated values in male subjects. Our model finding is consistent with a previous NCA analysis of ALB and ALB-OX in healthy individuals by Mirfazaelian A et al., in which systemic clearance and distribution of ALB-OX were lower in females (31). The oxidation reaction by cytochrome P450 (CYP450) enzymes is believed to mediate ALB-OX metabolism to the inactive metabolite albendazole sulphone (32). There are, however, almost no *in vitro* or *in vivo* studies that characterize the contribution of specific CYP-450 isoenzymes to ALB-OX metabolism. Sex-related differences in the activity of CYP-450 enzymes have been reported, and lower enzyme activity of specific CYP-450 isoenzymes in females might be responsible for the observed lower systemic clearance of ALB-OX compared to male subjects (33–35). However, future studies are needed to evaluate the impact of genotype and sex on ALB-OX metabolism.

A recently published PopPK analysis of ALB and ALB-OX in children and adults infected with soil-transmitted helminth infections showed that incorporating the impact of weight by allometric scaling on CL and VD parameters of ALB and height as a covariate on CL and VD of ALB-OX was associated with a significant improvement in model fit (23). In our analysis, height information was missing in 40% of the study population, limiting our ability to evaluate the impact of height or body weight-related measurements driven by height information, such as estimated fat-free mass or lean body weight. However, we do not anticipate that variations in body height will account for most of the IIV observed in our study population. Drug exposure data in our model were driven from the adult population compared to the model developed by Hofmann, Daniela et al. (23), where drug exposure was pooled from adults and children aged between 2–65 years. This wide age range could explain why height was a significant covariate in their analysis, which may be related to the possible correlation between growth-related measurements such as age and height and the maturation of body organs responsible for drug metabolism. In our analysis, the impact of body weight as a covariate or by allometric scaling did not significantly explain the associated IIV observed in our study population. In the Hofmann, Daniela et al. model, infection type also considerably affected the systemic clearance of ALB-OX, with patients infected with *Trichuris trichiura* showing a 26% increase in systemic clearance compared to those infected with hookworm. In a systematic review and modeling study by Whittaker C et al. (14), some parasitic infections, such as echinococcosis and neurocysticercosis, significantly impacted the PK of ALB-OX and systemic exposure. However, investigators found no statistically significant effect of LF infection on ALB-OX clearance or overall drug exposure (14). In our analysis, LF infection status did not explain the IIV in PK of ALB and ALB-OX, consistent with previous NCA findings on the impact of LF infection on ALB and ALB-OX exposure (10).

ALB’s absorption rate constant (Ka) showed significant IIV in our model, which could not be accounted for by the study subjects’ demographic and clinical characteristics. In all three clinical studies used for model building, ALB was administered with high-fat food; however, the amount of food consumed by subjects, as well as the percentages and types of fat, were not reported. The IIV observed in Ka could be correlated with possible variation in fat content and the amount of food consumed before ALB dosing. More standardization and control over food amount and type could help characterize food-associated variability in ALB exposure. However, in a bioequivalence study in which food amount and timing before ALB dosing were strictly controlled and standardized across study subjects, high IIV in the PK of ALB and ALB-OX was still observed (36).

The final model-based simulation revealed that virtual female subjects showed higher ALB-OX exposure than male subjects after a single 400 mg oral dose of ALB. The higher exposure in females was consistent with our covariate analysis finding, in which the systemic clearance of ALB-OX was lower in females than in males. In LF, studies investigating the correlation between exposure to preventive chemotherapy used in MDA, including ALB and ALB-OX, and filarial response in patients are limited. As a result, it is unclear whether the reduced ALB-OX exposure observed in our analysis is associated with a lower therapeutic response in males. Further *in vitro* and *in vivo* studies are required to evaluate the exposure-response relationship of ALB and ALB-OX, which will guide future dose recommendation studies.

There are a few limitations to this analysis. First, the VPC of the final model indicated underprediction of the lower 5th percentile of observed ALB concentrations in study subjects. This prediction misspecification may be due to ALB not being detected in plasma, particularly in samples collected 36, 48, and 72 h after dosing. ALB is essentially a prodrug for the active moiety ALB-OX. As such, ALB concentrations were below the LLOQ by 48 h for most participants. Analytical methods used to analyze ALB and ALB-OX plasma concentrations in clinical trials used for model building were highly sensitive, with LLOQ values lower than those of most other published methods (4, 36, 37). However, we do not anticipate that these VPC findings would impact the overall model prediction performance, since the model demonstrates a good ability to describe the central tendency of observed ALB concentrations. The second limitation of our study was the lack of pharmacogenomic data on the metabolic status of the study population. Liver enzymes mainly mediate ALB and ALB-OX metabolism, and including genotyping information regarding metabolism would have allowed us to investigate its influence on systemic clearance. In contrast to ALB-OX, ALB hepatic metabolism has been well characterized in *in vitro* and *in vivo* studies, with CYP3A4 and flavin-containing monooxygenase (FMO) mainly mediating its oxidation to ALB-OX (4, 38, 39). Future PopPK studies, including genotyping data for those two enzymes, will help understand the high IIV associated with ALB clearance and its impact on the metabolic formation of ALB-OX and its systemic clearance. Another limitation is the generalizability of the model’s findings to the pediatric population. In MDA programs for LF, children aged 2 years and older are eligible to receive the diethylcarbamazine plus albendazole regimen, whereas children aged 5 years and older are eligible for the IDA regimen; both regimens include ALB (40). However, the drug exposure data in our model were obtained through the administration of ALB to an adult population. As a result, our model’s findings should be interpreted with caution when applied to populations under 18 years of age, including for dosing simulations. Previously, model-based simulation showed that pediatric patients infected with hookworm had higher ALB-OX exposure than adults after simulated oral doses of ALB ranging from 100 mg to 800 mg (23). Higher exposure to ALB-OX in children may be related to age-related differences in the maturity of organs responsible for drug metabolism, particularly the liver. In our analysis, increasing age in adults was not associated with a significant impact on ALB or ALB-OX clearance. Future PopPK studies in LF-infected pediatric patients are needed to investigate the influence of age on ALB and ALB-OX clearance and, subsequently, overall drug exposure.

In conclusion, we developed a PopPK model to characterize ALB and ALB-OX exposure and investigate the reported high IIV in PK. To the best of our knowledge, this is the first PopPK analysis that utilizes drug exposure data from healthy individuals and LF patients residing in LF-endemic regions.
